# Molecular epidemiology of HIV-1 infection in immigrant population in northern Italy

**DOI:** 10.1017/S0950268819002012

**Published:** 2020-02-05

**Authors:** Caterina Sagnelli, Caterina Uberti-Foppa, Sabrina Bagaglio, Eleonora Cella, Vittoria Scolamacchia, Hamid Hasson, Stefania Salpietro, Emanuela Messina, Giulia Morsica, Silvia Angeletti, Massimo Ciccozzi, Adriano Lazzarin, Evangelista Sagnelli

**Affiliations:** 1 Department of Mental Health and Public Medicine, Campania University Luigi Vanvitelli, Naples, Italy; 2 Department of Infectious Diseases, Vita-Salute University, San Raffaele Scientific Institute, Milan, Italy; 3 Medical Statistics and molecular Epidemiology Unit, Campus Bio-Medico University, Rome, Italy; 4 Laboratory clinical Science, Campus Bio-Medico University, Rome, Italy

**Keywords:** HIV-1 subtype B, immigrants, molecular epidemiology

## Abstract

Human immunodeficiency virus-1 (HIV-1) is characterised by a vast genetic diversity classified into distinct phylogenetic strains and recombinant forms. We describe the HIV-1 molecular epidemiology and evolution of 129 consecutive HIV-1 positive migrants living in Milan (northern Italy). Polymerase gene sequences of 116 HIV-1 subtype-B positive patients were aligned with HIV-1 reference sequences (https://www.ncbi.nlm.nih.gov/) by using MAFFT alignment and edited by using Bioedit software. A maximum likelihood (ML) phylogenetic tree was performed by MEGA7 and was visualised by using FigTree v1.4.3. Of 129 migrants, 35 were born in Europe (28 in Eastern Europe), 70 in the Americas (67 in South America), 15 in Africa and nine in Asia; 76.4% were men who have sex with men (MSM). The serotype HIV-1-B prevailed (89.9%), followed by -C, -F1, -D and -A. Compared with 116 HIV-B patients, the 13 with HIV-non-B showed lower Nadir of CD4+ cell/mmc (*P* = 0.043), more frequently had sub Saharan origin (38.5 *vs.* 1.72%, *P* = 0.0001) and less frequently were MSM (40 *vs.* 74.5%, *P* = 0.02). The ML phylogenetic tree of the 116 HIV-1 subtype-B positive patients showed 13 statistically supported nodes (bootstrap > 70%). Most of the sequences included in these nodes have been isolated from male patients from the Americas and the most common risk factor was MSM. The low number of HIV-1 non-B subtype patients did not allow to perform this analysis. These results suggest a shift of HIV-1 prevention projects' focus and a continuous monitoring of HIV-1 molecular epidemiology among entry populations. Prevention efforts based on HIV molecular epidemiology may improve public health surveillance setting.

## Introduction

Two major types of human immunodeficiency virus (HIV) have been identified, HIV type 1 and HIV type 2 (HIV-2), with HIV-2, differing genetically from HIV-1 by nearly 55%. The rapid worldwide spread of HIV-1 has been favoured by its enormous genetic variability and rapid evolution, making the virus highly adaptable to new hosts. HIV-1 sequences differ by up to 10% in single individuals [1] due to its error-prone high replication and substitution rate consequent to the lack of proof-reading activity of the reverse transcriptase enzyme [2, 3]. HIV is classified into four distinct groups: M (major), O (outlier), N (non-M/non-O) and P [4–8]. Group M viruses are responsible for the HIV-1 global pandemic, further classified in distinct subtypes (A-D, F\H, J and K), sub-subtypes (A1, A2, F1 and F2) and circulating recombinant forms (CRFs) [‘www.hiv.lanl.gov/content/sequence/HIV/CRFs/CRFs.html'].

Subtype B is the dominant one in high-income countries with sexual transmissions among men who have sex with men (MSM), injection drug users (IDUs) and transfusions in hemophiliacs, with non-B subtypes/CRFs rising steadily among heterosexuals [9–15].

In Italy, the HIV-1 epidemic is mainly sustained by the subtype B genetic form, although other and novel subtypes and CRFs have been identified [16–19].

The percentage of infection with HIV-1 non-B subtypes ranged from 2.4% to 19.4% in different Italian studies reaching 63.0% among migrants [20–22], with an overtime significant increase [17, 23] paralleling the increase in migratory flows from geographic areas at high prevalence of HIV-1 infection [24–26]. Accordingly, the increased prevalence in HIV-1 non-B subtypes in Italy has been associated with the increase of the immigration flow [26, 27]. Indeed, the proportion of migrants among the new diagnoses of HIV infection has increased from 11% in 1992 to 35.8% in 2016 [‘http://old.iss.it/binary/publ/cont/COA.pdf’].

Ciccozzi *et al*. reported a 90.7% prevalence of HIV-1 subtype B in 215 HIV-1 *pol* gene sequences collected between 1992 and 2010, in Italy. Three main clusters were detected which roots dated to 1987. Most of the observed viral gene flow events occurred from heterosexual to intravenous drug users. Phylogenetic and molecular clock analysis showed an early HIV-1 subtype B introduction in the mid-1980 and dissemination within local risk-specific clusters [28].

Lo Presti *et al*. analysed 883 HIV-1 subtype pol gene sequences obtained from patients living in the geographical area of Bergamo (a chief town in northern Italy) declaring different transmission risks. In this study, the 25% of the observed gene flow was from people living in the north valleys to lowland and 40.5% from a heterosexual risk group to injecting drug users. The latter were identified as the central link and the mercenary sex as the most common route of transmission and gene flow between this group and both heterosexual and homosexual individuals [29].

The history of human being is history of migration which have determined the current ethnic composition of countries. At present, most of migrants come to north-western nations from their native countries in the south of the world for a variety of reasons, including family re-unification, desire for economic prosperity, natural disaster, dictatorial policy or escaping conflict. Due to the socioeconomic and political crises or tribal war in Sub-Saharan Africa counties, Western countries have become lands of immigration from this geographic area at high HIV endemicity. The migrant population living in Italy are prevalently male, young, sexually active [30] and with broken family ties. They often live in overcrowded residences, are not socially integrated and have limited access to healthcare services [31].

A high proportion of migrants living in European countries was born in geographic areas at high HIV endemicity and, consequently, is infected with HIV more frequently than the subjects born in the host country. The European Centre for Disease Prevention and Control reported that from 2007 to 2011 about 40% of all new cases of HIV infection diagnosed in Europe were registered in foreign patients [32], who nevertheless constituted about the 10% of the total resident population. In Italy, the median incidence of HIV infection at the end of 2013 was 19.1 per 100 000 for migrants and 4.9 per 100 000 for Italian-born subjects, reflecting the frequent arrival of migrants from sub Saharan countries [33].

Compared with the native populations of Italy and of other European countries, HIV infection is diagnosed in a more advanced stage in migrant populations [33–37], as was also confirmed in Italy by a recent survey [34], due to various psychological and bureaucratic barriers delaying HIV testing and antiretroviral therapy administration [38, 39].

The study of HIV-1 epidemic diversity provides a useful tool through which we can understand the history of the pandemic and monitor its spread and epidemic growth over time. The objective of this study was to analyse in detail the epidemiology, characteristics and evolution of HIV in 129 migrants residing in Milan (northern Italy). Since 116 of the 129 subjects were infected with HIV-1B and only 13 with HIV-1-non B, the phylogenetic and evolutionary analysis was focused on the HIV-1 subtype B sequences only.

## Material and methods

### Study population

One-hundred twenty-nine consecutive anti-HIV-1 positive adult migrants were enrolled from 2008 to 2018 at the Department of Infectious Diseases, Vita-Salute University, San Raffaele Scientific Institute, Milan, Italy.

The following criteria for including a patient into the study should have be fulfilled: (1) availability of a serum sample obtained at the first observation, stored frozen at −70 °C, never towed and used only for this investigation and (2) availability of registered demographic data, self-reported risk factors for the acquisition of HIV infection, geographic area of origin and biochemical virologic and immunologic data obtained at the time of first observation when all patients were naïve for anti-retroviral therapy. All the data mentioned above have been filled in a pre-coded questionnaire.

Criteria for exclusion were an ongoing or previous antiretroviral therapy and a not even slight compliance with the criteria required for inclusion (see above at points 1 and 2).

### Serological determinations

Serum HBsAg was sought by a commercial immunoenzymatic assay (Abbott Laboratories, North Chicago, IL, USA) and anti-HCV antibody by a 3rd generation commercial immunoenzymatic assay (Ortho Diagnostic Systems, Neckargemund, Germany). Antibodies to HIV 1 and 2 were sought using a commercial ELISA (Abbott Lab., North Chicago, Ill, USA) and positive results were always confirmed by Western blot analysis (Genelabs Diagnostics, Science Park Drive, Singapore), according to the Italian law.

Lymphocyte subsets (CD4+ and CD8+) were evaluated by flow cytofluorimetry using monoclonal antibodies and a fluorescence-activated cell sorter scan (Becton Dickinson, Mountain View, USA). Liver function tests, triglyceride and cholesterol levels were determined according to routine methods. The body mass index (BMI) was determined by standard procedures.

### Statistical analysis

Continuous variables were not normally distributed and were summarised as median and interquartile range, and categorical variables as absolute and relative frequencies. The Mann–Whitney and *χ*^2^ non parametric test have been used, the *P* value less than 5% was considered statistically significant. The statistical analysis was performed using Stata software version 14.1 (StataCorp Texas 77845 USA).

### Molecular epidemiology and phylogenetic procedures

For the phylogenic analyses only HIV-1 subtype B were considered (116 sequences on 129), because mostly represented.

Two different datasets were built to investigate the phylogenetic relationships and the genetic variability of the HIV-1 virus pol gene.

The first dataset was built using 116 HIV-1 virus pol gene subtype-B sequences plus 76 reference sequences, downloaded from NCBI [‘http://www.ncbi.nlm.nih.gov/genbank/’]. The second dataset were built using 33 HIV-1 virus pol gene subtype-B sequences. This dataset included sequences with patients known information and included in statistically supported clusters. These two datasets were used to perform, respectively, maximum likelihood (ML) and Bayesian dated trees, evolutionary and phylodynamic analyses.

The reference sequences were selected based on the following criteria: (1) sequences already published in peer-reviewed journals; (2) known sampling date and location and (3) all the available sequences as always described [40]. All sequences were aligned using MAFFT [41] and manual editing was performed with Bioedit, removing gaps and cutting to identical sequence lengths.

MEGA7 was used to select the simplest evolutionary model that adequately fitted the sequence data for the datasets by using the ‘Models’ tool. The phylogenetic signal was tested with TreePuzzle [42, 43], DAMBE [44] and MEGA7 [45] software.

The ML tree was inferred on the first dataset; the statistical robustness and reliability of the branching order was confirmed with the bootstrap analysis (bootstrap values >70%) [46, 47, ‘http://beast.bio.ed.ac.uk’].

The evolutionary rate of the HIV-1 virus pol gene subtype-B (second dataset) was estimated by calibrating a molecular clock using known sequence sampling times with the Bayesian Markov Chain Monte Carlo (MCMC) method implemented in BEAST v. 1.10.1 [48, 49]. To investigate the demographic history, independent MCMC runs were carried out enforcing both a strict and relaxed clock with an uncorrelated log normal rate distribution and one of the following coalescent priors: constant population size, exponential growth, non-parametric smooth skyride plot Gaussian Markov Random Field and non-parametric Bayesian skyline plot (BSP) [48–55]. Chains were conducted for at least 50 × 10^6^ generations and sampled every 5000 steps for each molecular clock model. Convergence of the MCMC was assessed by calculating the ESS for each parameter.

## Results

The initial characteristics of the 129 immigrants with HIV infection are reported in Tables 1 and 2. They aged 35.12 ± 8.65 years, have been followed-up for 9.59 ± 4.79 years and were prevalently males (82.94%). Reliable information on the main risk factor for acquiring HIV infection was available only for 110 patients; risky heterosexuality was declared by 21 (19.09%) of which two being sexual partner of an HIV-positive subject, male homosexuality by 84 (76.36%) of which four bisexual, drug addiction by four (3.64%) and being born of an anti-HIV positive mother by one (0.91%) (Table 1).

**Table 1.** Demographics and epidemiological characteristics of all the 129 HIV-1 positive immigrants enrolled, and according to HIV-1 serotype

	All cases	Serotype HIV-B	Serotype HIV-non-B	*P-*Value HIV-B *vs*. non B
No of patients	129	116	13	
Males, No (%)	107 (82.94)	99 (85.34)	8 (61.5)	0.7768
Age, years, (M ± s.d.)	35.12 ± 8.65	35.24 + 9.08	30.04 + 6.68	0.0293
Risk category, No. (%)				
Injective drug users	4 (3.63)	4 (3.96)	0	n.s.
Risky heterosexuality	21 (19.09)	16 (15.84)	5 (55.5)	0.0035
Male homosexuality	84 (76.36)	80 (79.20)	4 (44.4)	0.0211
Vertical transmission	1 (0.90)	1 (0.99)	0	n.s.
Missing information	19	15	4	
Geographical origin, no (%):				
Europe	35 (27.13)	32 (27.58)	3 (23.08)	0.8923
America	70 (54.26)	66 (56.89)	4 (30.76)	0.1062
Africa	15 (11.62)	9 (7.75)	6 (46.13)	0.0001
Asia	9 (6.98)	9 (7.75)	0	n.s.
HIV-1 serotype, No (%):		–	–	
A	1 (0.77)			
B	116 (89.9)			
C	6 (4.6)			
D	1 (0.77)			
F1	5 (3.9)

**Table 2.** Biochemical and immunovirological characteristics of the 129 HIV-1 positive immigrants, and according to HIV-1 serotype.

	All cases	Serotype HIV-B	Serotype HIV-non-B	*P*-Value HIV-B *vs*. non B
No of patients	129	116	13	
Males, No (%)	107 (82.94)	99 (85.34)	8 (61.5)	0.7768
Age, years, (M ± s.d.)	35.12 ± 8.65	35.24 ± 9.08	30.04 ± 6.68	0.0293
BMI, m^2^/kg, (M ± s.d.)	23.62 ± 3.61	23.60 ± 6.80	23.77 ± 4.7	0.9232
Nadir of CD4+, cell/mmc, (M ± s.d.)	326.58 ± 197.42	337.41 ± 196.16	230.77 ± 189.14	0.0430
CD4+, cell/mmc, (M ± s.d.)	472.00 ± 257.00	481.68 ± 265.47	393.92 ± 344.11	0.2360
CD4%, (M ± s.d.)	21.46 ± 9.3	21.85 ± 9.13	18.3 ± 10.28	0.1543
CD8+, cell/mmc, (M ± s.d.)	1118.00 ± 651.8	1106.6 ± 605.50	1199.62 ± 957.83	0.5970
CD8%, (M ± s.d.)	52.01 ± 12.7	51.21 ± 12.79	57.97 ± 10.12	0.0451
HIV-RNA, cps/ml, (M ± s.d.)	79 013.30 ± 171 001.37	76 509.54 ± 17 437.54	101 129.92 ± 142 109.75	0.0759
Creatinine, (M ± s.d.)	0.8 ± 0.15	0.78 ± 0.17	0.65 ± 0.16	0.0046
Bilirubin mg/dl, (M ± s.d.)	0.5 ± 0.2	0.51 ± 0.26	0.53 ± 0.31	0.7791
AST, IU/l, (M ± s.d.)	30.3 ± 16.53	36.37 ± 22.70	29.00 ± 12.2	0.2064
ALT, IU/l, (M ± s.d.)	35.6 ± 22.00	30.51 ± 16.95	28.9 ± 12.91	0.7159
GGT, IU/ml, (M ± s.d.)	45.98 ± 76.54	42.63 ± 62.98	73.08 ± 148.36	0.1468
ALP IU/ml, (M ± s.d.)	89.00 ± 92.3	81.5 ± 36.98	148.75 ± 258.27	0.0093
Glucose mg/dl, (M ± s.d.)	87.9 ± 16.4	87.70 ± 17.05	89.5 ± 10.12	0.6816
Triglycerides, mg/dl, (M ± s.d.)	122.4 ± 73.49	114.74 ± 76.77	93.67 ± 35.36	0.2827
Total cholesterol, mg/dl, (M ± s.d.)	166.57 ± 40.13	167.74 ± 41.22	157.25 ± 29.78	0.3285
HBsAg positive, N(%)	3 (2.33)	3 (2.58)	0	0.6520
HBsAb positive, N(%)	77 (59.7)	69 (59.48)	8 (61.53)	0.1098
Anti-HBc positive, N(%)	46 (35.66)	43 (37.07)	3 (7.69)	0.7946
Anti-HCV positive, N(%)	5 (3.87)	5 (4.31)	0	0.9900
HCV genotype, No (%):				–
1a	2 (40)	2 (40)		
2a	1 (20)	1 (20)		
2b	1 (20)	1 (20)		
4a	1 (20)	1 (20)		
Follow-up, years, (M ± s.d.)	9.59 ± 4.79	6.83 ± 4.8	9.45 ± 4.58	0.0420

Of the 129 migrants, 35 were born in Europe (28 in Eastern Europe), 70 in the Americas (67 in South America, of whom 21 in Brazil and 21 in Peru) and 15 in Africa (of whom seven born in sub Saharan Africa and nine in Asia) (Table 1).

The serotype HIV-1 B prevailed (89.9%), followed by serotypes HIV-1 C (4.6%), HIV-1 F1 (3.9%), HIV-1 D (0.78) and HIV-1 serotypes A (0.78%) (Table 1).

The 129 HIV-1 positive immigrants showed a quite good immunological condition at enrolment since the mean CD4+ cells per mm^3^ count was 472.00 ± 257.00 (M ± s.d.), the percentage of CD4% 21.46 ± 9.3 and the nadir median value through the observation 326.58 ± 197.42 CD4+ cell/mmc (Table 2). The mean HIV-RNA, cps/ml was 79 013.30 ± 171 001.37, indicating an active HIV replication (Table 2). In addition, the mean values of initial serum of liver enzymes, metabolic indexes and creatininemia and the infrequent coinfection with HBV (three cases) and with HCV (five cases) testify that the 129 patients as a whole had a satisfactory clinical condition (Table 2).

Compared with the 116 patients with serotype HIV-B, the 13 with serotype HIV-non-B showed a significantly lower Nadir of CD4+ cell/mmc (*P* = 0.043), more frequently were born in Sub-Saharan Africa (38.46 *vs.* 1.72%, *P* = 0.0001) and less frequently were MSM (40.0 *vs.* 74.5%, *P* = 0.02); no other significant demographic, epidemiological and laboratory difference was observed between these two subgroups of patients (Tables 1 and 2).

### Phylogenetic analysis

The best evolutionary model was K2 + G + I for the first and HKY + G for the second dataset. The phylogenetic noise for the datasets was investigated by means of likelihood mapping. The percentage of dots falling in the middle area of the triangles was 8.6% for the first dataset and 6.7% for the second dataset, as none of the dataset showed more than 30% of noise, contained sufficient phylogenetic signals.

The percentage of the Parsimony-Info sites ranged from 42% (the first) to 66.7% (second dataset); the percentage of the constant sites ranged from 23.84% (second dataset) to 46% (the first dataset).

The phylogenetic signal analysis using a transition/transversion *vs.* divergence graph and the Xia's test (*P* < 0.001) did not show evidence for substitution saturation. This indicated that enough signal for phylogenetic inference existed (data not shown).

All the analyses performed showed a sufficient phylogenetic signal for further phylogenetic analysis.

**Fig. 1.**
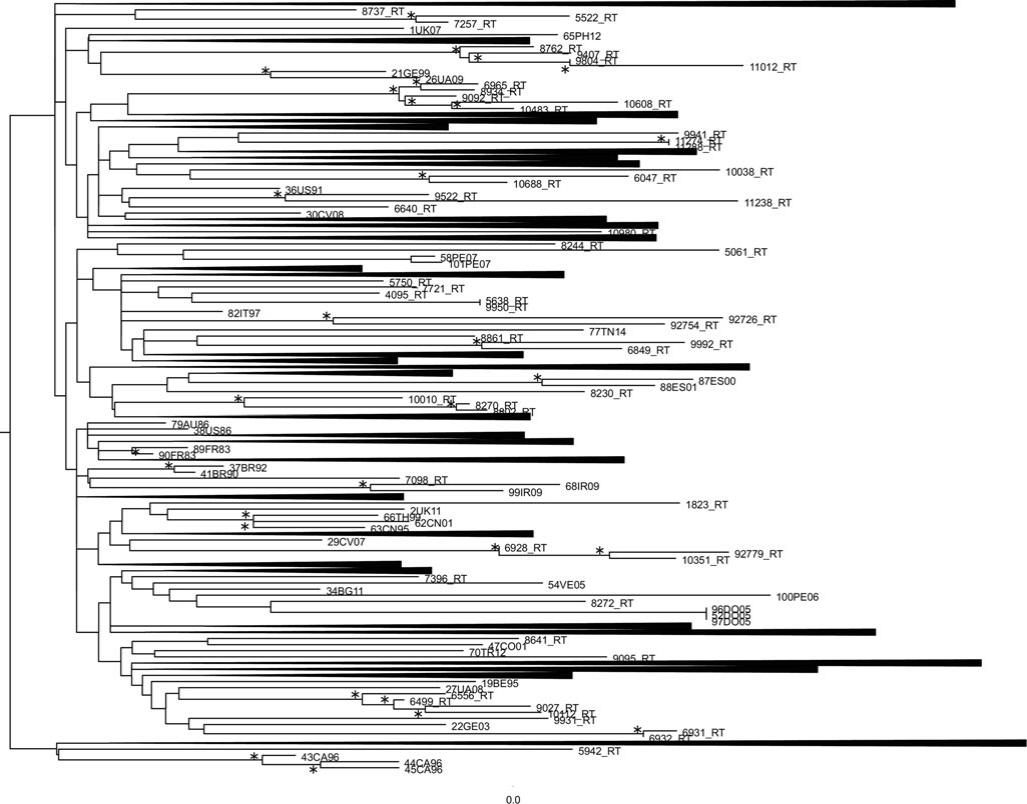
Phylogenetic relationship of the HIV-1 subtype-B sequences with the subtype-B reference sequences downloaded from the NCBI sequence database (https://www.ncbi.nlm.nih.gov/). The asterisk along a branch represents significant statistical support for the clade subtending that branch (bootstrap support 70%). The collapsed cluster in the figure were what not statistically significant.

ML phylogenetic tree of the first dataset was reported in Figure 1. Many statistically supported clusters have been found. The first cluster showed a correlation between a male patient born in Brazil tested for HIV-1 virus in 2008 and a heterosexual female patient born in Peru tested for HIV-1 virus in 2011. The second cluster underlined a relationship among a female patient born in Albania tested for HIV-1 virus in 2011, a female patient born in Albania, arrived in Italy in 2011, tested for HIV-1 virus in 2013 and a male patient born in Albania tested for HIV-virus in 2016. The third cluster is divided in two sub-clusters. The first sub-cluster showed a correlation between a homosexual male patient born in Montenegro tested for HIV-1 virus in 2008 and a homosexual male patient born in Germany tested for HIV-1 virus in 2011. The second sub-cluster showed a relationship among a homosexual male patient born in El Salvador tested for HIV-1 virus in 2012, a homosexual male patient born in Ecuador tested for HIV-1 virus in 2016 and a homosexual male patient born in Peru tested for HIV-1 virus in 2015. The fourth cluster observed a correlation between a male patient born in Spain tested for HIV-virus in 2016 and a male patient born in Peru, arrived in Italy in 2016, tested for HIV-1 virus in 2016. The fifth cluster showed a relationship between a homosexual male patient born in Ireland, tested for HIV-1 virus in 2008 and a homosexual male patient born in Philippines tested for HIV-1 virus in 2016. The sixth cluster evidenced a correlation between a homosexual male patient born in Brazil, arrived in Italy in 2009, tested for HIV-1 virus in 2012 and a homosexual male patient born in USA, texted for HIV-1 virus in 2016. The seventh cluster correlated a drug addicted female patient born in Nigeria tested for HIV-1 in 2013 and a HIV-1 virus subtype-B reference sequences isolated from Peru in 2007. The eighth cluster showed a relationship between a drug addicted female patient born in Nigeria tested for HIV-1 virus in 2013 and a male patient born in Tunisia tested for HIV-1 virus in 2014. The ninth cluster correlated the sequence 92726_RT and the sequence 92754_RT, two sequences with no information found. The tenth cluster showed a correlation between a heterosexual female patient born in Brazil tested for HIV-1 virus in 2014 and a homosexual male patient born in Colombia, arrived in Italy in 2008, tested for HIV-1 virus in 2009. The eleventh cluster supported a relationship between a homosexual male patient born in Turkey tested for HIV-1 in 2014, a homosexual male patient born in Ecuador tested for HIV-1 in 2009 and a homosexual male patient born in Peru for HIV-1 in 2011. The twelfth cluster showed a correlation among a homosexual male patient born in Peru tested for HIV-1 virus in 2008, the sequence 92779_RT with no information found and a homosexual male patient born in Philippines tested for HIV-1 in 2015.

The thirteenth cluster supported a relationship between a homosexual male patient born in Argentine tested for HIV-1 virus in 2010 and a homosexual male patient born in Bulgaria tested for HIV-1 virus in 2010. This cluster showed also a sub-cluster composed of a homosexual male patient born in Brazil tested for HIV-1 in 2012 and a homosexual male patient born in Spain tested for HIV-1 in 2014. The fourteenth cluster correlated a male patient born in Israel tested for HIV-1 virus in 2008 and a male patient born in Hungary and tested for HIV-1 in 2008.

### Bayesian reconstruction of the time-scaled phylogeny

**Fig. 2.**
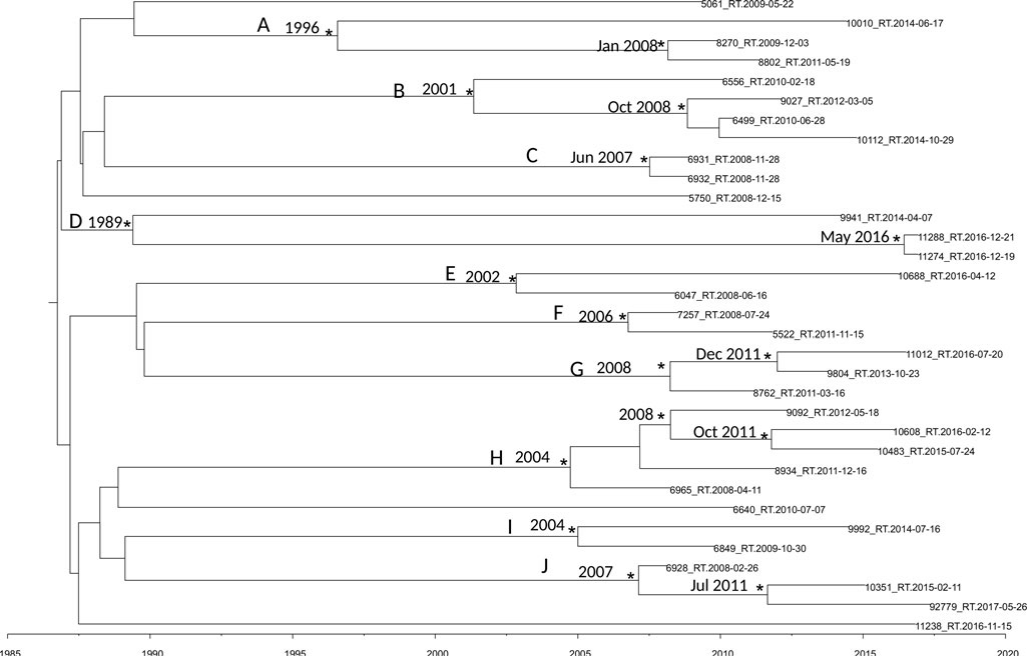
MCC tree of the second dataset. Branches are scaled in time. Significant posterior probability support (pp ≥ 0.9) as indicated by an asterisk. Clusters are highlighted.

Figure 2 shows the maximum clade credibility (MCC) tree of second dataset.

The BSP as a demographic model with a relaxed molecular clock was selected as the most appropriate to describe the evolutionary history of HIV-1 B pol sequences (lnBF > 6). Molecular clock calibration estimated the evolutionary rate of the HIV-1 B pol sequences at 2.17 × 10^−3^ substitutions site per year (95% HPD 1.37 × 10^−3^–2.9 × 10^−3^).

There were ten statistically supported clusters. Cluster A includes three sequences, isolated from male homosexual patients isolated in Turkey Ecuador and Peru. The most recent common ancestor (tMRCA) corresponding to 1996, inside this cluster two sequences formed a group dated back to January 2008.

Cluster B dated back to 2001 and included four sequences isolated from male MSM patients from Brazil, Argentina, Bulgaria and Spain.

Cluster C included two sequences, it dated back to June 2007. The virus of these two sequences was isolated in the same day and seems to be the same virus circulating in these two male patients.

Cluster D dated back 1989 and included three sequences isolated from one female and two males. Interestingly sequence 11288_RT was isolated from a male patient from Peru arrived in Italy in 2016 and the tMRCA of the cluster including this strain and one another sequences dated back to May 2016.

Cluster E included two sequences from male MSM patients, dating back to 2002.

Cluster F included two sequences from male and female patients, dating back to 2006.

Cluster G included three sequences from Albanian patients, dating back to 2008. Only of one strain was known the date of arrival in Italy, 9804_RT, the group that include this strain dated back to December 2011 and this patient arrived in Italy in 2011.

Cluster H included five sequences from male MSM patients, dating back to 2004.

Cluster I included two sequences from South-American patients, dating back to 2004, one of this two sequences belongs to a Colombian patient arrived in Italy in 2008.

Cluster J included three sequences from male patients, dating back to 2007; 92779_RT sequence was isolated from an Egyptian patient arrived in 2013 in Italy and the group that included this strain dated back in July 2011.

## Conclusion

It is commonly accepted that human mobility is linked to the transmission of, and to the susceptibility to infectious diseases. Migrants, defined as individuals who move from their country of origin to another, account for 40% of newly-diagnosed cases of HIV infection in the European Union/European Economic Area. Populations at high risk for HIV infection include migrants from countries where the HIV prevalence is high and those participating in high risk behaviour. Travellers contribute to the spread of HIV-1 genetic diversity worldwide, and in the developing world migration of rural populations and civil war are additional contributing factors.

Here we describe the characteristics and evolution of HIV-1 in immigrant population in northern Italy observed between 2008–2018. Phylogenetic analysis revealed different clusters with different characteristic factors probably causing the transmission of the virus within these groups. People mostly arrive in Italy already infected in their country of origin, sometime the transmission having occurred within the familiar marriage and sometimes, as in MSM and sex workers, because of frequent unsafe sexual intercourses. Unfortunately, in some cluster we did not catch any information to deduce an epidemiological link between phylogenetic tree results and the possible source of infection.

Migration is a humanitarian problem requiring political decisions including specific measures to reduce the number of susceptible people and improving their living condition. In this regard, analysing the measles model in a migrant population, some authors suggested screening and vaccination campaigns as the key points to achieve an adequate immunisation coverage and disease control [56]. A clinical surveillance performed in 2016 proved the good state of health of a migrant population, but the associated microbiological surveillance performed by blood, rectal, pharyngeal and nasal swabs traced unusual microorganisms such as Pseudomonas (P.) putida, P. monteilii, P. fulva, P. mosselii; Aeromonas (A.) veronii, A. caviae, A. hydrophila, A. guillouiae; Acinetobacter (Ac.) Ac. lwoffii, Ac. johnsonii, Ac. tjernberg; Pantoea (Pa.) agglomerans and Pa. calida. Among these microorganisms, some strains were resistant to carbapenems or ESBL producers or methicillin resistant [57]. Therefore, there is an indication to apply to migrant population measures useful to reduce the chances of transmitting infectious agents (screening and prophylaxis) from migrants to the native population, from this to migrants and among migrants.

The data presented in this study underline the importance of molecular and epidemiological screening for HIV infection for all newly arrived migrants, being they asylum seekers or economic migrants, since the spreading of HIV-1 variants has implications for diagnostic, treatment and vaccine development. These results suggest a shift of HIV-1 prevention projects' focus and a continuous monitoring of HIV-1 molecular epidemiology among entry populations. Prevention efforts based on HIV molecular epidemiology may improve public health surveillance setting.
